# tRNA Modifications and Dysregulation: Implications for Brain Diseases

**DOI:** 10.3390/brainsci14070633

**Published:** 2024-06-25

**Authors:** Xinxin Lv, Ruorui Zhang, Shanshan Li, Xin Jin

**Affiliations:** 1 School of Medicine, Nankai University, Tianjin 300071, China; 1910661@mail.nankai.edu.cn (X.L.); shanshan.li@nankai.edu.cn (S.L.); 2 Dana and David Dornsife College of Letters, Arts and Sciences, University of Southern California, Los Angeles, CA 90089, USA; oscarzha@usc.edu

**Keywords:** tRNA, brain, epilepsy, stroke, neurodevelopmental disorder, brain tumors, neurodegenerative disease

## Abstract

Transfer RNAs (tRNAs) are well-known for their essential function in protein synthesis. Recent research has revealed a diverse range of chemical modifications that tRNAs undergo, which are crucial for various cellular processes. These modifications are necessary for the precise and efficient translation of proteins and also play important roles in gene expression regulation and cellular stress response. This review examines the role of tRNA modifications and dysregulation in the pathophysiology of various brain diseases, including epilepsy, stroke, neurodevelopmental disorders, brain tumors, Alzheimer’s disease, and Parkinson’s disease. Through a comprehensive analysis of existing research, our study aims to elucidate the intricate relationship between tRNA dysregulation and brain diseases. This underscores the critical need for ongoing exploration in this field and provides valuable insights that could facilitate the development of innovative diagnostic tools and therapeutic approaches, ultimately improving outcomes for individuals grappling with complex neurological conditions.

## 1. Introduction

Neurological disorders represent a significant challenge to the medical and scientific communities due to their complexity and profound impact on human health. A deeper understanding of the molecular mechanisms underlying these disorders is essential for advancing diagnostic and therapeutic strategies. Recent studies have identified disruptions in transfer RNA (tRNA) as critical yet underexplored contributors to neurological pathologies.

tRNAs are traditionally recognized for their central role in protein synthesis, where they undergo numerous chemical modifications essential for their function. These modifications not only ensure the accuracy and efficiency of protein translation but also play crucial roles in regulating gene expression and maintaining cellular homeostasis. Emerging evidence suggests that aberrations in tRNA modifications contribute to the pathophysiology of various human diseases, suggesting their broader implications in cellular dysfunction.

In this review, we aim to synthesize recent advances in tRNA modification research, with a focus on their relevance to neurological disorders. By integrating insights from molecular biology, genetics, and clinical investigations, we highlight critical trends and discoveries that emphasize the role of tRNA modifications in neurological health and disease. Through this exploration, we seek to elucidate how altered tRNA modification contributes to the complexity of neurological disorders and potentially novel targets for therapeutic intervention.

## 2. Biological Processes of tRNA and Prevalent Modifications

### 2.1. Biological Processes of tRNA

#### 2.1.1. tRNA Transcription

**Figure 1:**
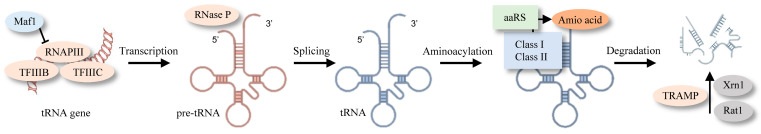
tRNA biogenesis pathway. RNAPIII: RNA polymerase III; TFIIIB, TFIIIC: transcription factors IIIB and IIIC; RNase P: ribonuclease P; aaRS: aminoacyl-tRNA synthetase; TRAMP: Trf4/Air2/Mtr4p polyadenylation complex; Xrn1, Rat1: exonucleases.

Transfer RNAs (tRNAs) are transcribed by RNA polymerase III (RNAPIII), a 17-subunit complex, while mRNAs are produced by RNA polymerase II [1,2]. RNAPIII also synthesizes crucial non-coding RNAs including 5S rRNA and U6 snRNA [3,4]. Transcription begins at tRNA gene promoter sites, A-box and B-box, essential for forming the tRNA’s structural arms [5,6]. This process is driven by transcription factors TFIIIC and TFIIIB, and RNAPIII’s C37 subunit, which initiates pre-tRNA synthesis by melting the promoter [7,8,9,10]. The newly formed pre-tRNAs may undergo oligouridylation at the 3′ end, with stability and proper folding ensured by the La protein [11,12,13,14]. Similar mechanisms are involved in the transcription of tRNASec and U6 snRNA, utilizing an internal B-box and an upstream element [15,16]. tRNA transcription is regulated by Maf1, which integrates signals from nutritional and stress conditions, modulating RNAPIII activity through its phosphorylation state [17,18,19]. This regulation ensures that tRNA synthesis aligns with cellular needs. The intricate network of transcription factors and their modulation underscore tRNA’s pivotal role in cellular function and adaptability. Research on tRNA components like selenocysteine has even led to potential new cancer therapies, demonstrating the broad implications of tRNA biology in health and disease [20] (Figure 1).

#### 2.1.2. tRNA Splicing and Processing

Pre-tRNA molecules undergo initial processing by RNase P, which removes the 5′ leader sequence [21,22,23,24], influenced by the phosphorylation state of the La protein at S366 [25]. The 3′ end of pre-tRNAs, rich in uridine, is trimmed by RNase Z in bacteria and ELAC2 in humans, while in eukaryotic cells, Rex1p and Rrp6p exonucleases, alongside the La protein, facilitate 3′ trailer removal [26,27,28,29]. For intron excision, the tRNA splicing endonuclease (TSEN) complex, consisting of subunits TSEN2, TSEN15, T of TSEN34, and TSEN54, accurately identifies and excises introns, a process taking place in the nucleus of mammalian cells and the mitochondria of yeast [30,31,32,33,34]. Exon rejoining is handled by tRNA ligases, with yeast utilizing Trl1 and vertebrates using the HSPC117 complex [35,36,37,38]. The final step involves the CCA-adding enzyme appending the CCA sequence essential for translation and export [39,40]. Uniquely, tRNA^His is modified by Thg1, which adds guanine at its 5′ end [41,42,43]. These complex splicing and processing steps are crucial for tRNA functionality and cellular adaptation, highlighting their significance in understanding molecular biology and disease pathology.

#### 2.1.3. tRNA Nuclear Transport

In eukaryotic cells, the transport of tRNA across the nuclear pore complex is tightly regulated [44,45]. Nuclear export of tRNA primarily utilizes β-importin family proteins, specifically Los1 in yeast and Exportin-t (Xpot) in vertebrates. These exportins operate in a Ran-GTP gradient-dependent manner to ensure directional movement of tRNA from the nucleus to the cytoplasm [46,47,48,49,50,51,52]. Specifically, Xpot binds to tRNA in the nucleus in its GTP-bound state, facilitated by Ran GTPase, with GTP hydrolysis upon transit releasing tRNA into the cytoplasm for participation in protein synthesis [53]. Additionally, pre-tRNAs requiring further splicing are exported via factors like Los1 and Msn5 to the TSEN complex at the mitochondrial outer membrane, demonstrating the intricate control over tRNA processing [54]. Conversely, mature tRNAs can re-enter the nucleus from the cytoplasm, particularly under stress or nutrient depletion, facilitated by proteins such as Mtr10 and Hsp70, reflecting adaptive tRNA localization in response to cellular conditions [55,56,57].

#### 2.1.4. Aminoacylation and Degradation of tRNA

Aminoacylation of tRNA is performed by aminoacyl-tRNA synthetases (aaRS), with 20 types corresponding to the 20 standard amino acids, divided into Class I and II based on catalytic site structures. This process is critical for ensuring the correct translation of the genetic code, requiring a precise match between tRNA and amino acids, particularly at the tRNA’s 3′ CCA end [58,59]. Modifications at this end can affect charging efficiency, while aaRS includes a proofreading function to maintain translational accuracy [60,61]. Disruptions in aminoacylation are linked to various diseases. Recent findings include a bacterial AmTARS and TLR2 interaction that modulates inflammatory responses in colitis, presenting potential therapeutic avenues [62]. During stress or nutrient deficiency, uncharged tRNAs activate the integrated stress response (ISR), highlighting the critical role of accurate aminoacylation in cellular health and disease management [63,64]. Innovations like split o-aaRSs provide new therapeutic strategies by facilitating gene translation modifications [65].

tRNAs are stable RNA molecules with a half-life of 2 to 3 days, attributed to their complex structures [66,67,68]. Despite their inherent stability, cells deploy specific pathways to degrade partially processed, hypomodified, or misfolded tRNAs, ensuring tRNA pool integrity. In the nucleus, the TRAMP complex identifies precursor and hypomodified tRNAs for degradation via the nuclear exosome [28,29]. The cytoplasmic rapid tRNA degradation (RTD) pathway targets mature, structurally compromised tRNAs using 5′-3′ exonucleases like Rat1 in yeast and Xrn1 in other eukaryotes [69,70,71,72]. Additionally, stress-induced cleavage by angiogenin produces tRNA-derived fragments, impacting protein synthesis, cell proliferation, and apoptosis [73,74]. These degradation mechanisms are crucial for maintaining cellular homeostasis and have significant implications for neurological health and disease management.

### 2.2. Diverse and Functional Modifications of tRNA

**Figure 2:**
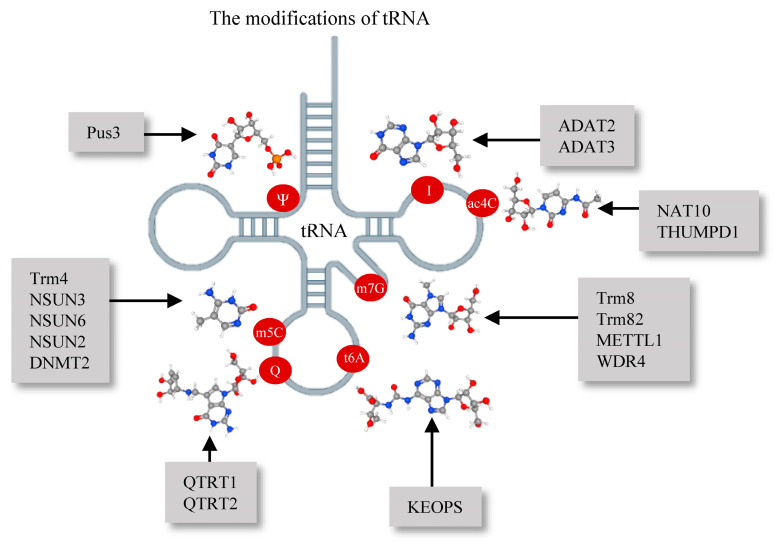
Modifications of tRNA and their enzymatic catalysts. Ψ: pseudouridine; I: inosine; ac4C: N4-acetylcytidine; m5C: 5-methylcytosine; m7G: 7-methylguanosine; t6A: N6-threonylcarbamoyladenosine; Q: queuosine; Pus3: pseudouridine synthase 3; ADAT2/ADAT3: adenosine deaminases acting on tRNA 2/3; NAT10: N-acetyltransferase 10; THUMPD1: THUMP domain-containing protein 1; Trm4: tRNA methyltransferase 4; NSUN: NOP2/Sun RNA methyltransferase family; DNMT2: DNA methyltransferase 2; Trm8/Trm82: tRNA methyltransferase 8/82; METTL1: methyltransferase-like 1; WDR4: WD repeat domain 4; KEOPS: kinase, endopeptidase and other proteins of small size complex; QTRT1/QTRT2: queuine tRNA-ribosyltransferase 1/2.

Recent studies have found over 200 different RNA modifications in various organisms, with tRNAs making up about half of them. These modifications involve various chemical processes like methylation, acetylation, deamination, isomerization, glycosylation, and pseudouridylation [75,76]. Different tRNA species have specific modifications, with an average of 13 per molecule. These modifications play various important roles in tRNA function beyond protein synthesis, such as maintaining the correct tRNA structure, ensuring accurate decoding of mRNA, enhancing translational fidelity, and regulating the interaction between tRNA and ribosomes [77,78]. Furthermore, tRNA modifications are important for cellular processes like growth, development, and stress response [79,80,81]. Notably, thermophilic organisms have unique tRNA modifications that adapt to changes in temperature, showing the critical role of these modifications in survival under extreme conditions [82] (Figure 2).

#### 2.2.1. m5C in tRNA

Methylation, the addition of a methyl group (CH_3_) to tRNA nucleotides, is crucial for structural stability and molecular interactions. In eukaryotic tRNAs, 5-methylcytosine (m5C) is commonly found at specific positions: 48, 49, 50 (between the variable loop and the T-ΨC loop), 34, 38 (in the anticodon loop), and 72 (in the acceptor stem). In Saccharomyces cerevisiae, Trm4 facilitates these modifications. In mammals, RNA cytosine methyltransferases NSUN3, NSUN6, NSUN2, and DNMT2 are involved, each targeting distinct tRNA species and positions [83,84,85]. Mutations in NSUN3 and NSUN2 are linked to mitochondrial encephalopathy, epilepsy, and neurodevelopmental disorders, emphasizing the importance of mt-tRNAMet methylation and m5C in neurodevelopment [84,85,86].

m5C modifications are essential for cellular defense against oxidative stress, enhancing cell survival by regulating stress-responsive protein translation. In mammalian cells, m5C protects tRNA from angiogenin-induced cleavage during stress. NSUN2 deficiency, leading to the absence of m5C, results in the accumulation of 5′ tRNA fragments that inhibit global translation initiation. This deficiency affects cell migration and adhesion protein production, significantly impacting neuronal cell migration and differentiation [87,88,89,90]. Additionally, defects in the Nsun2 gene reduce the abundance of specific tRNAs in tissues such as the skin, liver, and cerebral cortex. This reduction affects synaptic signaling proteins in the prefrontal cortex, influencing excitatory neurotransmission and behavioral outcomes [87,91,92,93,94,95].

#### 2.2.2. m7G Modification

The conversion of guanosine to 7-methylguanosine (m7G) at position 46 in tRNA is a conserved process in both eukaryotic and prokaryotic organisms, essential for maintaining tRNA structural integrity and functional efficiency [96]. In brewing yeast, this modification is catalyzed by the Trm8 and Trm82 complex, while in mammals, it is facilitated by proteins encoded by the METTL1 and WDR4 genes [97,98]. Mutations in WDR4 are associated with primordial dwarfism, characterized by microcephaly and intellectual development challenges, underscoring the critical role of m7G in neurodevelopment [96,99,100].

WDR4 is essential for cell growth and proliferation, with deficiencies potentially leading to early embryonic lethality [101,102]. Its activity is modulated by PKB kinase in the insulin signaling pathway, indicating a broader role in cellular regulation [103]. The m7G modification is crucial for maintaining tRNA folding and stability through precise hydrogen bond interactions [104]. Dysregulation of the Trm8 and Trm82 complex can lead to tRNA instability and degradation via the rapid tRNA degradation pathway, significantly impacting translation by affecting tRNA occupancy on ribosomes and mRNA translation efficiency [105]. Recent studies highlight the therapeutic potential of targeting m7G modifications. Manipulating tumor-intrinsic N7-methylguanosine tRNA modification can inhibit myeloid-derived suppressor cell (MDSC) recruitment, curb tumor proliferation, and enhance anti-PD-1 therapy effectiveness [106]. Additionally, targeting N7-methylguanosine tRNA modification shows promise in preventing hepatocellular carcinoma metastasis following inadequate radiofrequency ablation, offering a hopeful direction for cancer treatment [107].

#### 2.2.3. Pseudouridylation (Ψ)

Pseudouridylation, the enzymatic conversion of uridine to pseudouridine (Ψ), is a prevalent modification across diverse organisms, critical for tRNA functionality. In tRNAs, the 38th and 39th nucleotides in the anticodon stem-loop are commonly modified by pseudouridylate synthase 3 (Pus3) in Saccharomyces cerevisiae [108]. Pseudouridine (Ψ) enhances the thermal stability of the anticodon stem-loop by improving base-stacking interactions, a role observed in both yeast tRNA-Phe and human tRNA-Lys. This modification underscores the importance of pseudouridine in maintaining tRNA structural integrity [109,110,111]. The development of the reverse transcriptase-active DNA polymerase variant RT-KTq I614Y marks a significant advancement in RNA modification research. When paired with next-generation sequencing, this technology allows direct identification of pseudouridine and queuosine (Q) modifications in RNA without prior treatment, providing new insights into the effects of pseudouridylation on cellular mechanisms [112]. Yeast strains lacking Pus3 show increased protein aggregation and activation of stress response pathways, highlighting the enzyme’s crucial role [113,114]. In humans, homozygous inactivating mutations in the PUS3 gene are linked to developmental delays, intellectual disability, and reduced lifespan due to decreased Ψ levels in tRNA. These findings underscore the importance of PUS3 in neurodevelopment and cellular balance, particularly its role in conditions such as microcephaly and developmental delays, emphasizing its significance in human health and disease [115].

#### 2.2.4. t6A Modification

The N6-threonylcarbamoyladenosine (t6A) modification at the 37th position of tRNAs decoding ANN codons is a conserved feature across all domains of life [116,117,118]. This modification preserves the internal loop structure of tRNA through hydrogen bonding and π–π stacking interactions, preventing Watson–Crick pairing between U33 and A37 and stabilizing the anticodon stem-loop (ASL) [119,120,121]. t6A enhances the fidelity of codon–anticodon interactions within the ribosome, ensuring accurate positioning of tRNA with mRNA and facilitating the insertion of aminoacylated tRNA into the ribosome’s A site [122,123,124]. This modification is critical for efficient aminoacylation of tRNAs, optimizing translation efficiency, and maintaining cellular protein balance [125,126]. In eukaryotes and archaea, the KEOPS complex facilitates the t6A modification [127]. Mutations in KEOPS complex genes are linked to Galloway–Mowat syndrome, characterized by severe neurological and renal abnormalities, underscoring the significant impact of t6A on human health and development [128,129]. The absence of t6A can lead to translation errors, such as compromised initiation, faulty scanning of AUG codons by ribosomes, and increased initiation at non-AUG sites, resulting in protein misfolding, aggregation, and heightened cellular stress, all associated with neurological disorders [130,131].

#### 2.2.5. N4-Acetylcytidine (ac4C) in tRNA Function

The ac4C modification in Escherichia coli is primarily located at the wobble position of tRNA-Met, highlighting its crucial role in bacterial tRNA functionality [132,133]. In humans, N-acetyltransferase 10 (NAT10), in conjunction with THUMP domain-containing protein 1 (THUMPD1), facilitates the acetylation of serine and leucine tRNAs, reflecting a conserved mechanism across species [134,135,136,137]. Recent studies have linked THUMPD1 loss-of-function variants to a syndrome characterized by intellectual disability and developmental delays in 13 individuals from 8 families, underscoring the importance of ac4C in human neurodevelopment and cognitive function [138]. Situated in the D-stem region of serine and leucine tRNAs, ac4C promotes G base pairing, aiding in proper tRNA folding and facilitating crucial tertiary interactions [139,140]. Beyond its structural role, ac4C is vital in tRNA processing, aminoacylation, and accurate mRNA translation, emphasizing its essential function in protein synthesis [138]. Reduced NAT10 levels and subsequent decreases in ac4C-modified tRNAs significantly impact mRNA translation efficiency, highlighting the importance of tRNA modification in protein synthesis. Additionally, the identification of the epidermal growth factor receptor (EGFR) as a critical downstream target emphasizes NAT10′s role in cancer progression by modulating essential cellular pathways [141].

#### 2.2.6. The Role of Inosine in tRNA Function

The discovery of inosine as a critical modification in the anticodon loop of tRNA highlights its role in allowing tRNAs to recognize multiple synonymous codons. In eukaryotes, this modification occurs at the wobble position, facilitated by the adenosine deaminase complex ADAT2/ADAT3 [142,143]. Mutations in ADAT3 are linked to neurodevelopmental disorders, including strabismus, growth delay, intellectual disability, and microcephaly, underscoring the importance of precise tRNA modification for normal brain development and function [144]. Inosine can base pair with uridine, cytosine, or adenine, enabling a single tRNA isoacceptor to recognize multiple codons, thus enhancing translation fidelity and efficiency. This versatility is crucial in protein synthesis, where the thermodynamic and structural stability of inosine pairing is fundamental [145,146,147]. tRNAs with wobble inosines can translate codons ending in A more efficiently, reducing frameshift errors and affecting translation speed and preferences [148,149,150].

The abundance of inosine-containing tRNAs in eukaryotes suggests an evolutionary mechanism to enhance codon recognition and improve protein synthesis efficiency. This is supported by the prevalence of codons compatible with inosine-modified tRNAs in eukaryotic genomes, indicating a complex interaction between tRNA modifications and gene expression [151,152,153]. Inosine is essential for survival, with the ADAT2/3 complex playing a key role in tRNA decoding. Reduced inosine levels in species like Schizosaccharomyces pombe and Arabidopsis lead to growth and cell cycle issues, emphasizing its importance in maintaining cellular balance and efficient protein synthesis [143,154,155].

#### 2.2.7. Queuosine (Q)

Queuosine (Q) is a unique tRNA modification derived from queuine (G), characterized by a 7-deaza-guanine core and a cyclopentenediol structure. Located at the wobble base (34th position) of specific tRNAs, Q is essential for recognizing and interpreting NAY codons, integrating amino acids like tyrosine, histidine, aspartic acid, and asparagine in bacteria and eukaryotes [156,157]. In mammals, the gut microbiota supply queuosine, which is incorporated into tRNAs by the QTRT1-QTRT2 enzyme complex, demonstrating a symbiotic influence on gene expression. Queuosine at Q34 enhances translation accuracy and efficiency, ensuring protein synthesis fidelity. It prevents stop codon read-through in tRNATyr and modulates translation elongation rates, which are crucial for proper protein folding and proteome integrity [158,159,160,161,162,163,164]. The absence of Q modification reduces translation rates for Q-decoding codons, impacting overall translation dynamics, particularly in mitochondrial tRNAs where Q34 is essential for efficient UAU codon decoding [164,165]. Glycosylation of tRNAs with galactose and mannose affects translation rates and prevents stop codon read-through in vertebrates. Lack of Q glycosylation leads to increased protein aggregates, indicating Q’s role in maintaining protein balance [166,167]. Studies using a Qtrt1 gene knockout mouse model have linked the absence of Q-tRNA modification to learning and memory deficits, with more pronounced effects in female mice, suggesting potential sex-specific impacts on neurobiology [164].

## 3. The Role of tRNA in Brain Diseases

### 3.1. tRNA Dysregulation in Neurodevelopmental Disorders

**Figure 3:**
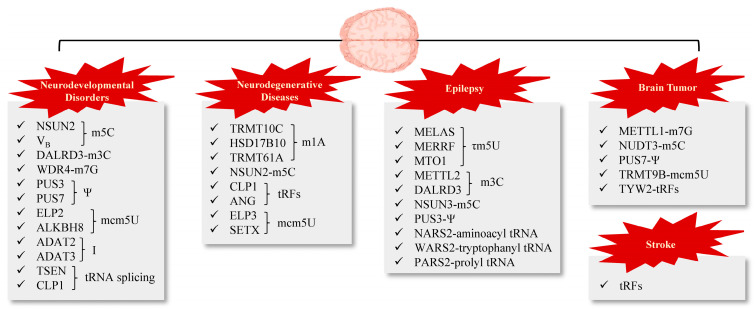
tRNA dysregulation and their associations with neurological disorders. The correlation between specific tRNA modifications and various brain diseases, including neurodevelopmental disorders, neurodegenerative diseases, epilepsy, brain tumors, and stroke. The implicated enzymes and modifications are highlighted for each condition.

Neurodevelopmental disorders commonly manifest during the prenatal period or shortly after birth, stemming from aberrant brain and nervous system growth and maturation. Recent studies have elucidated a notable correlation between these disorders and genetic mutations affecting tRNA modification, highlighting the crucial involvement of tRNA in normal brain development [168]. Changes in tRNA genes or those involved in their biosynthesis and modification have been increasingly associated with neurodevelopmental disorders. These modifications play a crucial role in neurodevelopment, where accurate and timely protein synthesis is essential. tRNA modifications help maintain precise codon–anticodon interactions, which are necessary for proper amino acid integration during protein synthesis. This process plays a crucial role in neuronal differentiation, synaptic plasticity, and overall brain function integrity. In addition to influencing protein synthesis, tRNA modifications impact ribosomal dynamics and translation rates, which are essential for brain development processes. This underscores the importance of studying the roles of tRNA modifying genes and enzymes in brain physiology and pathology (Figure 3).

NSUN2 enzyme is crucial for maintaining the stability and functionality of cytoplasmic and mitochondrial tRNAs through m5C modifications [88,93]. Mutations in NSUN2, a gene crucial for the m5C modification of tRNA, have been implicated in various neurodevelopmental disorders. These autosomal recessive variants manifest with a spectrum of symptoms, such as intellectual disability, microcephaly, behavioral abnormalities, delayed speech acquisition, distinctive facial characteristics, and growth impairment [85,169]. Research using Nsun2-deficient models demonstrates that aberrations in NSUN2-mediated methylation render tRNAs vulnerable to angiogenin-induced cleavage, leading to the accumulation of tRNA-derived fragments (tRF-5) in the brain. This accumulation impairs neuronal differentiation and responsiveness to growth factors, culminating in pronounced neurodevelopmental impairments [87,89]. Further studies indicate that NSUN2 deficiency triggers a stress response, characterized by elevated apoptosis in critical neuronal regions, including the cortex, hippocampus, and striatum. Interventions targeting oxidative stress, like inhibiting angiogenin, could help reduce the effects of Nsun2 loss. Studies in mice show that altering tRNA methylation levels, either by depleting NSUN2 or overexpressing it, can impact depression and cognitive function, linking tRNA modifications to emotional regulation [94].

The absence of DALRD3 in human cells, which is crucial for the m3C modification in tRNA^Arg^, results in severe neurological conditions such as early-onset epileptic encephalopathy and pronounced developmental delays. This genetic deficiency emphasizes the essential role of m3C modification in maintaining the functional integrity of the neural system [170]. Similarly, tRNA modifications involving m7G are critical for neural differentiation and brain development. Mutations in the WDR4 gene, which are pivotal for m7G modifications, lead to significant neurological disorders, including primary microcephaly and severe growth retardation. This highlights the importance of m7G in regulating genes essential for neural lineage development [100,171]. Additionally, the presence of biallelic mutations in the structural domain of ALKBH8, associated with tRNA modification processes, correlates with syndromic forms of intellectual disability. This relationship further illustrates the complex link between specific tRNA modifications and cognitive development, suggesting a direct connection between molecular alterations and neurological impairments [172].

The PUS3 gene, which encodes pseudouridine synthase-3, plays a critical role in the tRNA modification process, especially affecting pseudouridylation at positions 38 and 39 [173]. Recent findings have associated novel homozygous truncating mutations in PUS3 with intellectual disability, demonstrating the crucial role of pseudouridylation in cognitive development. Individuals with these mutations show significantly reduced levels of pseudouridylation in their tRNAs, leading to severe neurodevelopmental conditions, including global developmental delays, epilepsy, hypotonia, and microcephaly [173,174]. Furthermore, mutations in the PUS7 gene have been linked to specific neurodevelopmental challenges, such as language developmental delays and aggressive behaviors. Studies using Drosophila models have shed light on how PUS7 mutations directly contribute to these behavioral deficits, broadening our understanding of the impact of tRNA modifications on neurological and developmental disorders [175].

The eukaryotic elongator complex, initially known for its involvement in transcriptional elongation by interacting with hyper-phosphorylated RNA polymerase II, is now predominantly acknowledged for its contribution to tRNA modification. Originally composed of three core subunits—ELP1, ELP2, and ELP3—the primary focus of the complex has transitioned from regulating transcription to improving translational accuracy by modifying tRNAs. ELP2 plays a significant role in tRNA modification by catalyzing the transformation of uridine (U) into 5-methylcytidine (mcm5) and 5-methylcytidine-2-thiolate (mcm5s2). The function of ELP2 is crucial for preserving neuronal health and facilitating proper brain development. Dysfunction or impairment in ELP2 activity is associated with marked neurodevelopmental anomalies, potentially culminating in disorders characterized by intellectual disability and autism spectrum disorders. This association underscores the critical role of ELP2-mediated tRNA modifications in underpinning neurodevelopmental processes and sustaining cognitive and behavioral functions [176]. Mouse models of familial dysautonomia (FD), linked to Elp1 mutations, demonstrate ER stress and UPR, indicating the elongator complex’s involvement in various neurodevelopmental phenotypes, including intellectual disability (ID) and autism spectrum disorders [177,178].

In human cells, the conversion of adenosine to inosine at the wobble position of tRNA, catalyzed by the ADAT2/ADAT3 complex, constitutes a critical modification process. This alteration significantly enhances the versatility and accuracy of protein synthesis by broadening codon recognition capabilities. Recent findings have unveiled novel pathological variants of the ADAT3 gene that compromise its adenosine deaminase functionality. These variants, discovered within a family with a history of intellectual disability, involve critical missense mutations or premature stop codons within the deaminase domain, resulting in reduced inosine levels in tRNA and diminished adenosine deaminase activity. These genetic alterations highlight the intricate link between tRNA modification pathways and cognitive development [179]. The identified ADAT3 variants interfere with the interaction with the ADAT2 subunit and impact the complex’s nuclear localization, emphasizing the crucial role of these proteins in the nuclear import mechanism and the maintenance of tRNA inosine modification levels. Remarkably, experimental overexpression of the ADAT2 catalytic subunit in cells derived from affected individuals restored normal tRNA adenosine deaminase activity and inosine modification levels. This breakthrough not only elucidates the functional synergy within the ADAT2/ADAT3 complex but also paves the way for exploring therapeutic strategies to counteract ADAT3 deficiencies [179].

Human transfer RNAs (tRNAs) may contain introns that are crucially excised by the tRNA splicing endonuclease (TSEN) complex. This tetrameric enzyme is essential for preserving tRNA molecule integrity and functionality and is pivotal for precise protein synthesis [180,181]. Mutations in TSEN subunits and the related RNA kinase, CLP1, have been associated with pontocerebellar hypoplasia (PCH), highlighting the indispensable role of tRNA splicing in neurodevelopment. PCH is characterized by cerebellum and pons underdevelopment, often accompanied by microcephaly, profound motor disorders, and early childhood mortality [182,183].

Folate, an essential B vitamin, plays a pivotal role in neural development, influencing cytosine-5 methylation (m5C) in tRNA and affecting translation within mammalian mitochondria. Recent research has highlighted the critical impact of folate levels on mRNA methylation patterns in neural stem cells (NSCs), establishing a direct connection between folate availability and gene expression essential for brain development [184]. The complex relationship between tRNA modifications and neurodevelopmental outcomes has generated interest in investigating tRNA modification pathways as novel therapeutic targets. Through modulation of gene expression associated with tRNA modification, researchers aim to identify potential avenues for addressing neurodevelopmental disorders, ushering in a new era of therapeutic strategies.

### 3.2. The Emerging Role of tRNA Dysregulation in Neurodegenerative Diseases

Neurodegenerative diseases, including Alzheimer’s disease, Parkinson’s disease, and progressive supranuclear palsy, are distinguished by the gradual deterioration of neuronal function and cognitive abilities. Although protein aggregation, mitochondrial dysfunction, and oxidative stress have traditionally been recognized as key factors in their development, recent attention has turned to the complex involvement of tRNA modifications.

In the context of Alzheimer’s disease, a prevalent form of dementia, recent research has unveiled notable modifications in tRNA, particularly in the N1-methyladenosine (m1A) alterations, which are acknowledged for their impact on tRNA structural integrity and correct folding [185]. Studies utilizing the 5XFAD mouse model of Alzheimer’s disease have revealed a decrease in m1A methylation levels in mitochondrial and cytosolic tRNAs compared to control groups. Additionally, a reduction in the expression of enzymes involved in m1A modification in mitochondrial (TRMT10C, HSD17B10) and cytosolic (TRMT61A) tRNAs has been documented. This decrease in enzyme expression has been associated with more severe phenotypes in Drosophila models of tauopathy, indicating a significant correlation between alterations in m1A methylation and mitochondrial tRNA expression, potentially contributing to the pathogenesis of the disease [186]. Additionally, research conducted by Silzer et al. has identified significant hypermethylation at site 9 of mt-tRNA in cerebellar tissues from patients with progressive supranuclear palsy and Alzheimer’s disease [187]. These observations suggest that dysregulation in m1A modification, particularly the hypo-methylation of mt-tRNA, may be a common pathological feature across neurodegenerative diseases. Such insights open new avenues for understanding the molecular basis of these complex conditions.

A comprehensive examination of small RNA sequencing data obtained from key brain regions, specifically the nucleus accumbens (NAc), has elucidated a significant reduction in CholinotRFs (tRNA fragments originating from the mitochondrial genome) within the NAc of individuals with Alzheimer’s disease. This decline is strongly associated with the gradual deterioration of cholinergic activity and the advancement of cognitive decline. Additionally, the investigation highlights distinct gender-specific variations in the expression of cholinergic transcripts in Alzheimer’s disease, emphasizing the pivotal role of CholinotRFs in modulating cholinergic function [188]. The investigation of tRNA modifications presents potential avenues for therapeutic interventions and provides a fresh perspective on comprehending and treating neurological disorders.

Findings from the sticky (sti) mutant mice have provided clarification on the negative effects of impaired tRNA charging on neural health. In particular, deficiencies in appropriately charged tRNA^Ala^ result in the buildup of misfolded proteins in cerebellar neurons, ultimately leading to cell death. These results suggest the importance of tRNA synthetase proofreading and precise tRNA charging in preventing neurodegeneration, indicating that compromised translation accuracy, through mischarged tRNAs, could potentially contribute to neuronal harm and degeneration. [189]. Furthermore, modifications to tRNA that alter its stability or structural conformation can significantly influence the biogenesis of tRNA-derived fragments (tRFs) and their subsequent interactions with intracellular targets.

tRFs are small RNA molecules generated from precursor or mature tRNAs, generated from the specific cleavage of precursor and mature tRNAs by nucleases such as angiogenin, Dicer, RNaseZ, and RNase1. These fragments can be classified into several types based on their origins within the tRNA molecule, such as 5′-tRFs, 3′-tRFs, and internal tRFs. tRFs have been implicated in various biological processes [190,191], including gene regulation, cellular stress responses, and the modulation of protein synthesis [192]. The altered expression profiles of tRFs under neurological stress and disease conditions underline their significant regulatory roles in neurological disorders such as stroke, Alzheimer’s disease, epilepsy, Parkinson’s disease, and others, indicating their potential as biomarkers and therapeutic targets [193]. In neurodegenerative disease, models such as the Nsun2 RNA-MTase mutant mouse have demonstrated that increased levels of tRFs correlate with reduced protein translation. This observation implicates tRFs in the pathogenesis of conditions characterized by decreased brain size and neural signaling anomalies. Specifically, the loss of the m5C modification, mediated by NSUN2, leads to angiogenin-mediated tRNA cleavage, resulting in the accumulation of 5′ tRFs associated with microcephaly and other neurological impairments [87,194]. Beyond their association with diseases, tRFs have been shown to play protective roles in embryonic development, particularly in inhibiting the transposition of endogenous retroviruses in mice. Their dysregulation, especially in the context of CLP1 kinase mutations, results in significant neurodevelopmental defects and neurodegeneration. Furthermore, variations in the angiogenin gene (ANG) have been implicated in several neurodegenerative diseases, with specific tRF subgroups offering predictive insights into the outcomes of epilepsy and Parkinson’s disease [182,195,196,197,198,199,200].

The elongator complex, a dodecameric assembly, plays a crucial role in acetylating the wobble uridines (U34) of 11 tRNA species, a process essential for maintaining optimal translation rates and proteome integrity. Hypomodification of elongator-dependent tRNAs disrupts protein homeostasis, potentially leading to protein misfolding and aggregation, a hallmark of neurodegenerative conditions [201,202]. Elongator subunit 3 (ELP3) has been identified as a critical factor in modulating the progression of neurodegenerative diseases, notably Amyotrophic Lateral Sclerosis (ALS). In zebrafish models carrying mutations in SOD1 and C9orf72, linked to ALS, ELP3 expression mitigated axonopathy, indicating a neuroprotective function of ELP3 through tRNA modification. This effect was corroborated in the SOD1G93A ALS mouse model, where ELP3 expression extended survival and alleviated motor neuron denervation. The correlation between ELP3 expression, tRNA modification (specifically mcm5s2U), and the solubility of mutant SOD1 proteins emphasizes the importance of ELP3-mediated tRNA modifications in understanding neurodegenerative disease mechanisms [203]. In ALS patients, reduced expression of ELP3 in the motor cortex correlates with decreased levels of the modified tRNA wobble uridine mcm5s2U and an increased presence of insoluble SOD1, TDP43, and FUS proteins. These observations suggest that dysregulation of the elongator complex impacts the pathology of neurodegenerative diseases, including ALS axonopathy [204]. Furthermore, mutations in elongator complex subunits and related genes, such as SETX, are associated with a spectrum of neurodegenerative diseases, endoplasmic reticulum (ER) stress, and the unfolded protein response (UPR).

### 3.3. The Role of tRNA Dysregulation in Brain Tumor Pathogenesis

tRNA is not only essential for protein synthesis but also significantly contributes to the epigenetic modulation of gene expression in tumors, affecting various cellular functions such as proliferation and stress response mechanisms. This highlights its potential significance in the initiation, progression, and intricate biology of cancer. [205]. Central nervous system (CNS) tumors, while uncommon, have a substantial impact on cancer-related morbidity and mortality. These encompass both primary tumors originating within the brain or spinal cord and secondary tumors that have metastasized from other regions of the body. Among primary brain tumors, gliomas, including low-grade gliomas (LGGs) and glioblastomas (GBMs), are notably prevalent and severe in adult populations, with GBMs representing the most aggressive subtype [206,207,208].

Bioinformatics analyses have revealed a correlation between the dysregulation of N7-methylguanosine (m7G) methylation and the progression of glioma, with RNA methyltransferase 1 (METTL1) being identified as a key factor. METTL1, which is responsible for m7G modification, has been recognized as a potential biomarker for glioma [209]. Elevated levels of METTL1 expression have been linked to increased proliferation of glioma cells and have the potential to impact the mitogen-activated protein kinase (MAPK) signaling pathway. Furthermore, the association between heightened METTL1 expression and unfavorable prognosis indicates its potential as an autonomous risk factor in gliomas, emphasizing its importance in tumorigenesis and resistance to therapies such as temozolomide [210,211]. The study conducted by Wang et al. revealed variations in the expression of m7G regulatory factors in glioma tissues compared to non-tumorous controls, specifically showing an increase in DCPS, NUDT1, NUDT5, and NUDT3 and a decrease in CYFIP2, LARP1, EIF4G3, and NCBP2. These differential expression profiles are closely linked to the malignant nature of glioma, indicating a substantial relationship between m7G RNA modification and the severity of glioma [212,213]. Moreover, the variance in expression levels of DCPS, EIF4E1B, NUDT1, and NUDT16L1 between glioblastomas (GBMs) and low-grade gliomas (LGGs) suggests their involvement in the progression of glioma malignancy [214].

The methyltransferase NSUN3 plays a critical role in facilitating the m5C modification of mitochondrial tRNA, thereby increasing energy production through enhanced protein synthesis in the mitochondrial respiratory chain. This modification is essential for promoting cancer cell invasion and metastasis, underscoring the significance of mitochondrial tRNA modifications in modulating cancer cell metabolism and metastatic capabilities. Nevertheless, the potential impact of inhibiting NSUN3 or mitochondrial protein synthesis using antibiotics as a potential therapeutic approach to mitigate cancer cell aggressiveness warrants further investigation due to potential repercussions on normal cellular functions and immune responses [215].

Elevated expression and catalytic activity of pseudouridine synthase 7 (PUS7) have been observed in glioblastomas, correlating with unfavorable patient prognoses. The significant involvement of PUS7 in promoting tumor growth in glioblastoma stem cells (GSCs) underscores its potential as a crucial therapeutic target. The use of chemical inhibitors to disrupt PUS7 function presents a promising avenue for halting tumor advancement and prolonging survival in glioblastoma models, presenting a novel approach to combatting this aggressive form of brain cancer [216]. In contrast, the tumor suppressor protein ADAR2, known for its protective role in aggressive brain tumors, is antagonized by ADAR3, a fellow member of the ADAR protein family. The intricate interaction among ADAR family proteins in brain malignancies underscores the intricate nature of post-transcriptional regulation in oncogenesis and presents a specific target for further therapeutic investigation [217]. 

The discovery of TRMT9B as a novel regulatory factor in synaptic formation underscores its potential significance beyond tumor suppression. With its structural resemblance to yeast tRNA methyltransferase 9 (Trm9) responsible for methylating tRNA wobble uridines, the involvement of TRMT9B in synaptic functionality underscores the broad implications of tRNA modifications in neural health and disease, including their impact on tumor biology [218].

The efficacy of current treatment protocols for gliomas, encompassing surgery, radiotherapy, and chemotherapy, is constrained, particularly in cases of glioblastoma multiforme (GBM), highlighting the imperative for novel diagnostic and therapeutic strategies [219,220]. Recent research has recognized tRNA-derived small fragments (tRFs) as potential biomarkers and therapeutic modalities for gliomas. Significantly, the tsRNA originating from tRNA-Cys-GCA, in conjunction with tRFdb-3003a and tRFdb-3003b, demonstrates marked downregulation in glioma tissues. Particularly noteworthy is the inhibitory effect of tRFdb-3003a on glioma cell proliferation and tumor growth in an in vivo setting. The interplay between tRFdb-3003a/b and the VAV2 protein, as well as their combined influence on glioma advancement, introduces novel therapeutic avenues. Furthermore, the correlation between their expression levels and patient survival rates presents a promising prognostic tool [221]. In glioblastoma multiforme (GBM), the most aggressive type of primary brain tumor, certain transfer RNA fragments (tRFs), specifically tRF-19-R118LOJX and tRF-19-6SM83OJX, exhibit significant differences in expression levels between low-grade glioma and GBM, and are associated with unfavorable clinical outcomes. These tRFs have the potential to act as inhibitors of glioma cell proliferation, migration, and angiogenesis, with tRF-19-R118LOJX targeting the 3′ untranslated region (3′UTR) of S100A11 mRNA, suggesting a promising therapeutic approach [222]. The alteration of 4-demethylwyosine (imG-14) assumes importance in the realm of chemoresistance, particularly in the context of agents such as paclitaxel. Diminished TYW2 enzyme expression results in elevated imG-14 levels, promoting cancer cell viability and bolstering resistance to paclitaxel. The targeting of imG-14 synthesis presents a potential avenue for circumventing this resistance, thereby presenting a novel approach in the field of cancer treatment [223].

Research is currently underway to uncover the impact of tRNA modifications on the molecular pathogenesis of gliomas and other central nervous system tumors. These modifications have been shown to influence tumor cell metabolism, protein synthesis efficiency in stressful environments, and the ability of cancer cells to evade immune surveillance or withstand treatment. A comprehensive comprehension of the functions and mechanisms of tRNA modifications in gliomas presents exciting opportunities for the identification of novel diagnostic indicators and therapeutic targets, which could ultimately result in improved treatment strategies for malignant brain tumors.

### 3.4. The Emerging Role of tRNA Dysregulation in Epilepsy

Epilepsy, a commonly occurring neurological disorder, is distinguished by repetitive seizures stemming from abnormal neuronal discharges. The initiation of this condition encompasses a multitude of neuropathological mechanisms, such as ion channel malfunction, neuronal injury, inflammatory responses, changes in synaptic plasticity, and proliferation of glial cells. Despite extensive research, the exact causes of epilepsy are still unknown, presenting a significant challenge in neurology. Recent progress in genetic and molecular studies has revealed the importance of tRNA modifications in epilepsy pathogenesis.

Mutations in mitochondrial tRNA (mt-tRNA) genes and dysregulation of tRNA-modifying enzymes have been recognized as potential contributors to heightened susceptibility to epilepsy. These modifications play a crucial role in tRNA functionality, impacting protein synthesis and mitochondrial stability. Perturbations in these pathways may predispose individuals to neurological disorders such as epilepsy by influencing neuronal excitability and energy metabolism [224]. Mitochondrial disorders such as MELAS and MERRF are associated with compromised mt-tRNA modification. Specifically, deficits in modifying the first anticodon nucleotide 5-taurinomethyluridine (τm5U) and its 2-thiouridine derivative (τm5s2U) in mt-tRNALeu(UUR) are pivotal in these diseases’ pathogenesis [225,226]. The reduced τm5U and τm5s2U modifications disrupt the efficient decoding of codons crucial for mitochondrial respiratory chain protein translation, impairing mitochondrial functionality and contributing to neurological symptoms, including epilepsy. The correlation between deficiencies in tRNA modification and mitochondrial dysfunction underscores the intricate nature of mitochondrial disorders and underscores the critical role of adequate tRNA modification in maintaining mitochondrial function and mitigating the risk of epilepsy.

Research has shown that high doses of taurine can alleviate defects in the modification of mitochondrial tRNA^Leu(UUR)^ observed in peripheral blood leukocytes, suggesting its potential as a therapeutic strategy to alleviate epilepsy symptoms in patients. This approach highlights the therapeutic importance of targeting tRNA modifications to enhance mitochondrial function and combat epilepsy [227]. Enhanced progress has been made through the upregulation of MTO1, a tRNA modification enzyme, in cells derived from individuals with MELAS syndrome. This intervention effectively reinstated the modification of mitochondrial tRNAs, resulting in enhanced mitochondrial function. Moreover, Mtu1 (Trmu), a critical tRNA modification enzyme, plays an essential role in the τm5s2U modification at position 34 of tRNAs, such as tRNA^Lys^, tRNA^Glu^, and tRNA^Gln^, which are vital for protein synthesis. These enzymes are necessary for preserving the precision of mitochondrial protein synthesis and functionality [228]. These findings provide a foundation for the development of targeted therapeutic interventions aimed at specific tRNA modifications in the treatment of mitochondrial diseases such as MELAS and MERRF. By investigating the epitranscriptomic regulation of mitochondrial tRNAs, novel therapeutic modalities are being elucidated, presenting promising prospects for the implementation of effective treatments that target the root causes of these disorders.

In mammals, the METTL2 methyltransferase, in collaboration with the DALRD3 protein, specifically targets a subset of arginine tRNA isoacceptors for methylation in the anticodon loop, resulting in the formation of 3-methylcytidine (m3C). This precise modification process is impaired in human cells lacking DALRD3, leading to an almost complete absence of m3C modification. Remarkably, homozygous nonsense mutations in DALRD3 have been associated with developmental delay and early-onset epileptic encephalopathy, underscoring the critical need for m3C modification in normal brain development [170].

NSUN3 functions as an m5C methyltransferase, specifically targeting the “wobble” position 34 of mt-tRNA^Met^. This modification pathway, which includes the oxidation of m5C to f5C by ALKBH1, is crucial for mitochondrial function. Disruptions resulting from the loss of NSUN3 or ALKBH1 lead to compromised mitochondrial translation, and decreased cell proliferation, and have been linked to early-onset mitochondrial encephalomyopathy and seizures. Notably, mutations in NSUN3, such as c.421G>C (p.A141P) and c.454T>A (p.C152S), have been associated with these severe neurological conditions [83,84,229,230]. PUS3, recognized for its role in modifying various RNAs including tRNA, is identified as a critical enzyme in neurodevelopment. Analysis of 21 individuals with PUS3 variants uncovered biallelic mutations as a rare but consequential cause of neurodevelopmental disorders, including epilepsy. This evidence underscores the importance of studying the function of PUS3 and tRNA modifications in elucidating the processes of brain development and neurological disorders [173].

Recent findings have identified two novel missense variants in the NARS2 gene in a patient with early-onset epilepsy and cardiac abnormalities. The patient’s clinical profile included early-life seizures, developmental delays, and cardiac dysfunction, which evolved into intractable epilepsy, further developmental challenges, and hyperlactatemia. These findings underscore the crucial role of NARS2 in neurological and cardiac health, indicating that mutations in aminoacyl-tRNA synthetases like NARS2 can disrupt normal brain and heart development, leading to severe epilepsy and systemic conditions [231]. Furthermore, mutations in the WARS2 gene, responsible for encoding tryptophanyl-tRNA synthetase 2, have been correlated with a variety of neurological manifestations, such as epilepsy and motor deficits. The epilepsy phenotypes linked to WARS2 mutations encompass a spectrum of conditions, ranging from developmental and epileptic encephalopathies (DEE) observed in neonates or infants to less clearly defined seizure disorders. The condition of DEE is distinguished by its early onset and seizures that frequently prove challenging to manage, thereby increasing the likelihood of progressing to status epilepticus and potentially leading to mortality. These findings illuminate the substantial influence of WARS2 mutations on neurological development and function, underscoring the varied and frequently severe manifestations of epilepsy linked to deficiencies in tRNA synthetase enzymes [232].

Pathogenic biallelic mutations in the mitochondrial prolyl-tRNA synthetase 2 gene (PARS2) have been identified as causative factors for a specific subtype of developmental and epileptic encephalopathies (DEE) characterized by sleep-related spikes. This syndrome, referred to as developmental epileptic encephalopathy with sleep-related spikes (DEE-SWAS), establishes a connection between epileptic abnormalities and gradual cognitive deterioration. The identification of PARS2 as the causative gene for DEE-SWAS highlights the genetic diversity underlying this syndrome and underscores the essential role of mitochondrial tRNA synthetases in the etiology of complex neurological conditions [233]. Additionally, mutations in the neuron- and muscle-specific translation factor eEF1A2, specifically G70S, E122K, and D252H, have been implicated in epilepsy. These mutations impair protein synthesis, leading to altered neuronal morphology by demonstrating increased affinity for tRNA and reduced affinity for actin. The direct impact of these mutations on the translation mechanism provides insight into how disruptions in protein synthesis and cytoskeletal interactions contribute to the development of epilepsy, further delineating the molecular pathways involved in epileptogenic processes [234].

In instances of neuronal stress, tRNA molecules may undergo cleavage to generate tRNA fragments (tRFs), which serve a dual purpose by potentially interfering with typical protein synthesis and contributing to the regulation of stress responses. This dual functionality establishes tRFs as significant contributors to the modulation of neuronal excitability and the initiation of seizures. Recent studies have established a correlation between distinct modifications in tRFs present in plasma and epilepsy, specifically refractory focal epilepsy. Of significance, levels of three tRF-5 variants (Gly-GCC, Ala-TGC, and Glu-CTC) in plasma have been observed to rise before seizure onset in individuals with epilepsy, indicating a responsive reaction to epileptic events, followed by a gradual return to levels comparable to those of healthy individuals after the seizure episode [199]. The production and excretion of transfer RNA fragments (tRFs) by neurons, particularly in the setting of neuronal hyperexcitability, demonstrate a complex cellular reaction to epileptiform activity. The reduction in the secretion of tRFs derived from 5′Gly-GCC and 5′Glu-CTC following epileptic events implies the initiation of defensive or responsive brain mechanisms, which may provide new perspectives on the mechanisms governing seizure control and the involvement of tRFs in the pathology of epilepsy.

The research conducted by Hazel et al. [235] presents a novel electrochemical detection approach employing electrocatalytic platinum nanoparticles to measure distinct tRNA fragments (tRFs) such as 5′AlaTGC, 5′GlyGCC, and 5′GluCTC. This technique showcases exceptional sensitivity and selectivity in identifying tRFs within limited blood samples, suggesting potential advancements in the creation of non-invasive diagnostic instruments for epilepsy. These advancements improve our capacity to monitor seizure activity and neuronal stress using blood-based biomarkers, representing a significant advancement in the non-invasive evaluation of epilepsy.

### 3.5. The Role of tRNA Dysregulation in Stroke

Stroke, a predominant cause of disability and death worldwide, is classified into ischemic and hemorrhagic types. Ischemic stroke, constituting the bulk of stroke incidents, arises from cerebrovascular issues such as arterial constriction or occlusion. This obstruction hampers cerebral blood flow, leading to tissue injury and neurological impairments [236,237]. Recent research reveals that stroke patients exhibit increased levels of tRNA-derived fragments (tRFs) in their plasma compared to healthy individuals. The presence and levels of these tRFs are significantly correlated with the degree of cerebral damage, relating to the size of the infarct in ischemic strokes and the volume of the hematoma in hemorrhagic strokes. These observations suggest that stress-induced tRFs could serve as biomarkers for evaluating cerebral damage and might predict the functional outcomes in stroke patients [238]. tRFs are implicated in important biological processes that are integral to the pathogenesis of stroke, including apoptosis, oxidative stress response, microglial activation, and angiogenesis. The involvement of tRFs in these pathways underscores their potential utility not only as biomarkers but also as targets for therapeutic intervention to modulate the course of stroke rehabilitation. Accurate identification of stroke types is crucial for proper treatment, as different strokes require specific therapeutic approaches. tRNA-derived fragments are valuable biomarkers for distinguishing stroke subtypes, improving diagnosis accuracy and treatment effectiveness [239].

Recent investigations have highlighted significant changes in the expression of tRNA-derived fragments (tRFs) following stroke incidents, suggesting a regulatory role for tRFs that could influence various signaling pathways essential for neuronal recovery post-stroke. Identifying the specific mRNA targets of these altered tRFs has elucidated their role in modulating key processes involved in post-stroke healing [239,240]. A critical analysis conducted by Li et al. identified six distinct tRNA-derived fragments (tRFs) that exhibit differential expression in response to intracerebral hemorrhage (ICH) conditions. Specifically, five tRFs (rno-tRF5-Glu-29a, rno-tRFi-Glin-16a, rno-tRF5-Ala-16a, rno-tRFi-Leu-35a, rnon-tRFi-Ser-25a) were found to be downregulated, while one tRF (rno-tRFi-Cys-20a) was upregulated. These tRFs are associated with various pathways relevant to ICH recovery, such as embryonic morphogenesis, endocytosis, the oxidative stress response, and G protein-coupled receptor signaling [241]. These pathways are implicated in neurodevelopment and neuroregulation post-intracerebral hemorrhage (ICH), suggesting a potential involvement of transfer RNA fragments (tRFs) in neural plasticity and recovery. Endocytosis, which is associated with microglial phagocytosis, plays a crucial role in the absorption of hematoma and restoration of tissue homeostasis following ICH [242,243]. Oxidative stress, known to compromise the blood-brain barrier and induce central nervous system (CNS) dysfunction, suggests that tRFs may contribute to preserving mitochondrial function and neural integrity [244].

The therapeutic potential of tRNA-derived fragments (tRFs) is increasingly recognized in the context of traditional Chinese medicine, specifically Bu Yang Huan Wu Decoction (BYHWD), which is known for its benefits in neurological recovery after stroke. BYHWD has been associated with neuroprotection, neurogenesis, and enhanced synaptic plasticity following stroke. Certain tRFs, such as rno-tRFi-Ser-25a, rno-tRF5-Glu-29a, and rno-tRF5-Ala-16a, show decreased expression levels after intracerebral hemorrhage (ICH) but exhibit restored levels following BYHWD treatment. The observed downregulation of their target transcripts post-BYHWD treatment suggests a regulatory role for these tRFs in critical pathways, including Foxo1 and IL-17 [245,246,247,248]. Cao et al. have discovered two distinct RNA fragments, namely FAM-tRF-His-GTG and tRFGln-CTG, which have been associated with cytotoxicity by inducing ribosomal stalling and inhibiting protein synthesis. This mechanism may result in neuronal demise, marked by elevated glutamate release, mitochondrial dysfunction, and eventual onset of stroke. The identification of these tRFs highlights the possibility of modulating their expression as a means to mitigate the risk of stroke, offering a fresh perspective on stroke management and prevention [249].

During the acute phase of large vessel occlusion, the plasma concentrations of tRNA-derived fragments, specifically tRNA-derived stress-induced RNAs (tiRNAs), exhibit potential for early detection of cerebral injury. tiRNAs are generated from tRNAs in response to cellular stress, particularly under conditions such as oxidative stress, hypoxia, and nutrient deprivation [74,190]. These fragments arise from the cleavage of mature tRNAs at the anticodon loop, resulting in the formation of 5′- and 3′-tiRNAs [250]. Furthermore, the generation of tiRNAs, facilitated by vascular endothelial growth factors in response to cellular stress, is recognized as a pivotal indicator in the pathophysiology of stroke [251,252]. In rat models of focal cerebral ischemia-reperfusion (I/R) injury, levels of tiRNA were observed to increase post-injury but were notably diminished after treatment with minocycline, a neuroprotective intervention, indicating their potential efficacy in assessing the efficacy of stroke therapies [251,253]. Subsequent investigations underscore the critical function of Maf1 in the control of Pol III-mediated rRNA and tRNA synthesis in murine neurons, particularly through its interactions with the mTOR signaling cascade. The manipulation of Maf1 activity has been shown to influence neuronal development and dendritic spine morphogenesis, with decreased levels promoting growth and increased levels hindering it. In the aftermath of a cerebral infarction, Maf1 levels rise in the peri-infarct region, and diminishing its expression has been found to enhance neuronal adaptability and promote functional recuperation. These findings indicate that Maf1 may serve as a potential therapeutic target for promoting neural repair and functional recovery following stroke and neural injuries [254].

## 4. Conclusions

This comprehensive review examines the diverse roles of tRNA modifications and rRNA dysregulation in brain diseases, highlighting their critical involvement in the pathophysiology of neurodevelopmental disorders, neurodegenerative diseases, brain tumors, epilepsy, and stroke. We underscore the necessity for additional research to unravel the intricate mechanisms of tRNA modifications and their associations with neurological disorders. Discovering new biomarkers and potential therapeutic targets within these pathways offers hope for the development of improved diagnostic methods and treatments. Ultimately, a deeper understanding of tRNA modifications and their implications in neurological disorders may lead to novel strategies for managing and treating these complex diseases, thereby enhancing health outcomes for individuals affected by a wide range of neurological conditions.
